# Gate-controlled generation of optical pulse trains using individual carbon nanotubes

**DOI:** 10.1038/ncomms7335

**Published:** 2015-02-27

**Authors:** M Jiang, Y Kumamoto, A Ishii, M Yoshida, T Shimada, Y. K. Kato

**Affiliations:** 1 Institute of Engineering Innovation, The University of Tokyo, Tokyo 113-8656, Japan

## Abstract

In single-walled carbon nanotubes, electron–hole pairs form tightly bound excitons because of limited screening. These excitons display a variety of interactions and processes that could be exploited for applications in nanoscale photonics and optoelectronics. Here we report on optical pulse-train generation from individual air-suspended carbon nanotubes under an application of square-wave gate voltages. Electrostatically induced carrier accumulation quenches photoluminescence, while a voltage sign reversal purges those carriers, resetting the nanotubes to become luminescent temporarily. Frequency-domain measurements reveal photoluminescence recovery with characteristic frequencies that increase with excitation laser power, showing that photoexcited carriers provide a self-limiting mechanism for pulsed emission. Time-resolved measurements directly confirm the presence of an optical pulse train synchronized to the gate voltage signal, and flexible control over pulse timing and duration is also demonstrated. These results identify an unconventional route for optical pulse generation and electrical-to-optical signal conversion, opening up new prospects for controlling light at the nanoscale.

## Abstract (web summary)

The photocurrent and luminescence of carbon nanotubes is governed by excitonic processes with diverse uses in nano-photonics. Here, Jiang *et al.* generate optical pulses from individual air-suspended carbon nanotubes under an application of square-wave gate voltages with control over pulse timing and duration.

## Graphical abstract

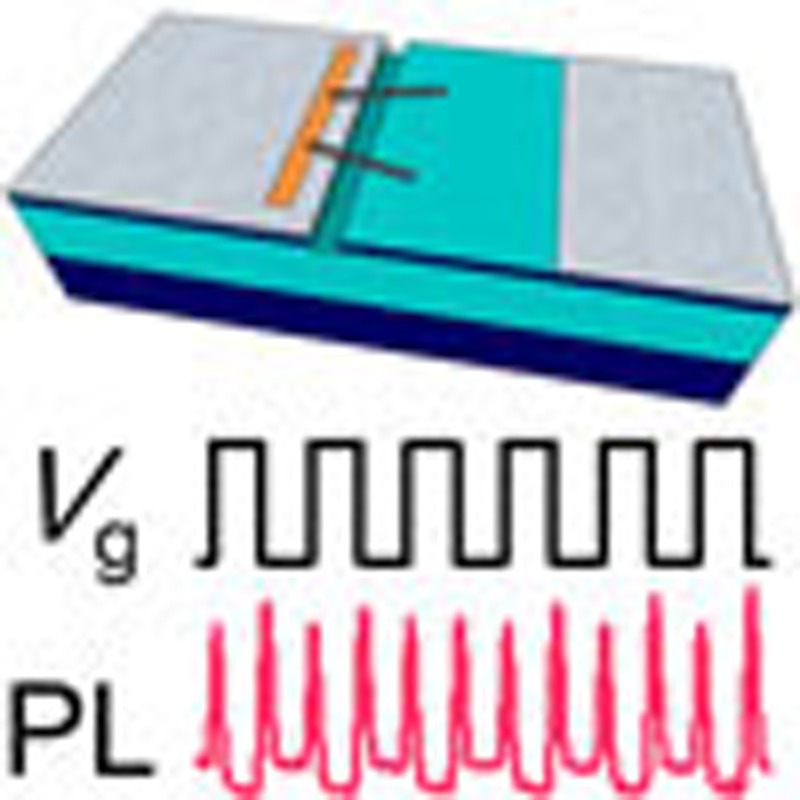

Being telecom-band emitters^1^,^2^ that can be directly synthesized on silicon^3^, single-walled carbon nanotubes (CNTs) are an appealing material for nanoscale optical devices. The limited screening of Coulomb interaction in one-dimensional systems^4^ leads to excitons with large binding energies^5^,^6^,^7^ that make them stable even at room temperature, and these excitons dominate the optical properties of CNTs. Unique excitonic processes give rise to complex photocurrent and electroluminescence mechanisms^8^,^9^,^10^,^11^ in CNTs, further offering diverse opportunities in nanoscale photonics and optoelectronics. In particular, interplay between free carriers and excitons is known to play an important role in determining the emission efficiencies^12^,^13^. It has been shown that carrier-mediated Auger recombination results in photoluminescence (PL) quenching under electrostatic^14^,^15^ and chemical doping^16^, but investigation of its dynamical response has remained elusive.

Here we investigate the luminescence response of individual CNTs subjected to square-wave gate voltages and unexpectedly find that such exciton–carrier interactions result in optical pulse-train generation. By performing experiments in both frequency and time domains, we show that the voltage transitions can temporarily purge photocarriers to generate self-limiting optical pulses. Our results demonstrate flexible control over pulse timings and widths, expanding the possibilities for optoelectronic circuits using CNTs^17^.

## Results

### PL recovery under square-wave gate voltages

**Figure 1:**
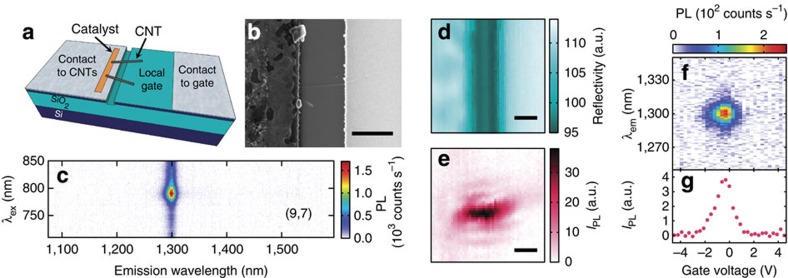
Device characterization. (**a**) Schematic of a device. (**b**) Electron micrograph of a device. (**c**) PL excitation map of a nanotube in a device measured in (**d**–**g**). (**d**) Reflectivity image taken with *λ*_ex_=761 nm. (**e**) PL image of the same area as **d**. (**f**) PL spectra as a function of gate voltage, taken at *P*=1 μW. (**g**) Gate voltage dependence of *I*_PL_. The slight offset of the peak from 0 V is likely caused by water adsorption^35^. In **b**,**d**,**e**, scale bars are 1 μm. (**c**–**e**) are taken with *P*=20 μW and (**e**–**g**) are taken at *λ*_ex_=790 nm. The spectral integration window for *I*_PL_ is from *λ*_em_=1,275 to 1,325 nm.

A schematic of our device is shown in Fig. 1a. Air-suspended CNTs are contacted on one side of a trench, and a local gate on the opposite side is used for applying electric fields onto the CNTs. By performing nanotube growth at the last step, we are able to take advantage of the superior optical properties of as-grown suspended nanotubes^18^,^19^,^20^, while the use of the top silicon layer of a silicon-on-insulator substrate allows for efficient and fast gating. An electron micrograph of a nanotube in a device is shown in Fig. 1b.

We characterize the nanotubes using PL microscopy^21^. Figure 1c shows PL as a function of excitation wavelength *λ*_ex_ and emission wavelength *λ*_em_ taken with a laser power *P*=20 μW, from which the chirality (9, 7) is assigned^22^,^23^. By comparing the reflectivity image (Fig. 1d) to the PL image obtained by mapping out the integrated PL intensity *I*_PL_ (Fig. 1e), we confirm that the tube is fully suspended. The d.c. gate voltage characteristics of the device is shown in Fig. 1f,g. On application of a gate voltage *V*_g_, PL quenching occurs as a result of phase-space filling and doping-induced exciton relaxation^12^,^13^,^14^,^15^,^16^.

**Figure 2:**
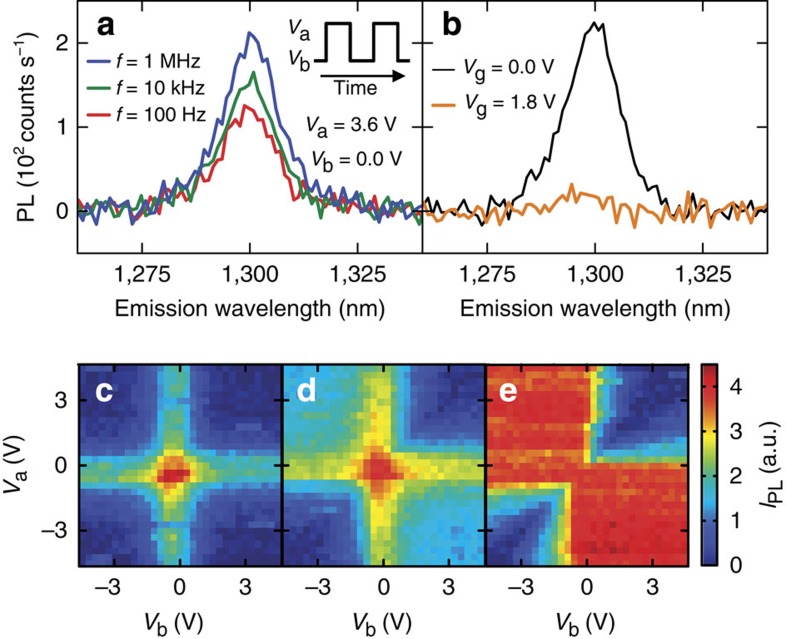
Square-wave gate voltage-induced PL recovery. The device characterized in Fig. 1c–g is measured with *P*=1 μW. (**a**) PL spectra taken under square-wave voltage with *V*_a_=3.6 V and *V*_b_=0.0 V at *f*=100 Hz (red), 10 kHz (green) and 1 MHz (blue). Inset is a schematic showing the definitions of *V*_a_ and *V*_b_. (**b**) PL spectra taken with d.c. voltages of *V*_g_=0.0 V (black) and *V*_g_=1.8 V (orange). (**c**–**e**) Integrated PL as a function of *V*_a_ and *V*_b_ at (**c**) *f*=100 Hz, (**d**) 10 kHz and (**e**) 1 MHz. The PL integration window is from *λ*_em_=1,275 to 1,325 nm.

Interestingly, we find that such quenching can be eliminated at high frequencies when square-wave gate voltages are applied. As shown in the inset of Fig. 2a, we use a waveform that alternates between *V*_a_ and *V*_b_ at a frequency *f*. The PL spectra taken with *V*_a_=3.6 V and *V*_b_=0.0 V for various *f* are shown in Fig. 2a. At *f*=100 Hz, the PL intensity is about half the intensity of that taken at *V*_g_=0.0 V (Fig. 2b, black curve). This is expected as PL is quenched for half of the time. As the square-wave frequency becomes higher, however, the emission intensity increases, and it recovers to the zero-voltage level at *f*=1 MHz (Fig. 2a, blue curve). Imaging measurements confirm that the recovered emission originates from the same nanotube (Supplementary Fig. 1).

The observed PL recovery cannot be due to time averaging of the gate voltage at high frequencies, as the capacitive cutoff frequency of the device is estimated to be larger than 50 MHz (Supplementary Note 1). In fact, we observe significant quenching for a static field corresponding to the time-averaged voltage (Fig. 2b, orange curve).

To investigate the mechanism underlying the PL recovery, we have measured the integrated PL intensity as a function of *V*_a_ and *V*_b_ for three different frequencies. At *f*=100 Hz (Fig. 2c), the PL intensity behaves as expected from the d.c. characteristics. PL is brightest when *V*_a_=*V*_b_=0 V, and quenching is observed when both *V*_a_ and *V*_b_ are non-zero. Along the lines that correspond to *V*_a_=0 V and *V*_b_=0 V, the PL intensity is approximately half of that for *V*_a_=*V*_b_=0 V, resulting in the cross shape in the *V*_a_−*V*_b_ map.

As the frequency is increased to *f*=10 kHz, we find that the PL starts to recover in the top-left and bottom-right areas in the map (Fig. 2d). These areas correspond to the cases where the signs of *V*_a_ and *V*_b_ are opposite, suggesting that PL recovery takes place through a process occurring at a voltage transition passing through 0 V. At *f*=1 MHz, the PL intensity in those two areas in the *V*_a_−*V*_b_ map becomes uniform, indicating that the recovery is complete (Fig. 2e).

### Frequency dependence and microscopic mechanism

**Figure 3:**
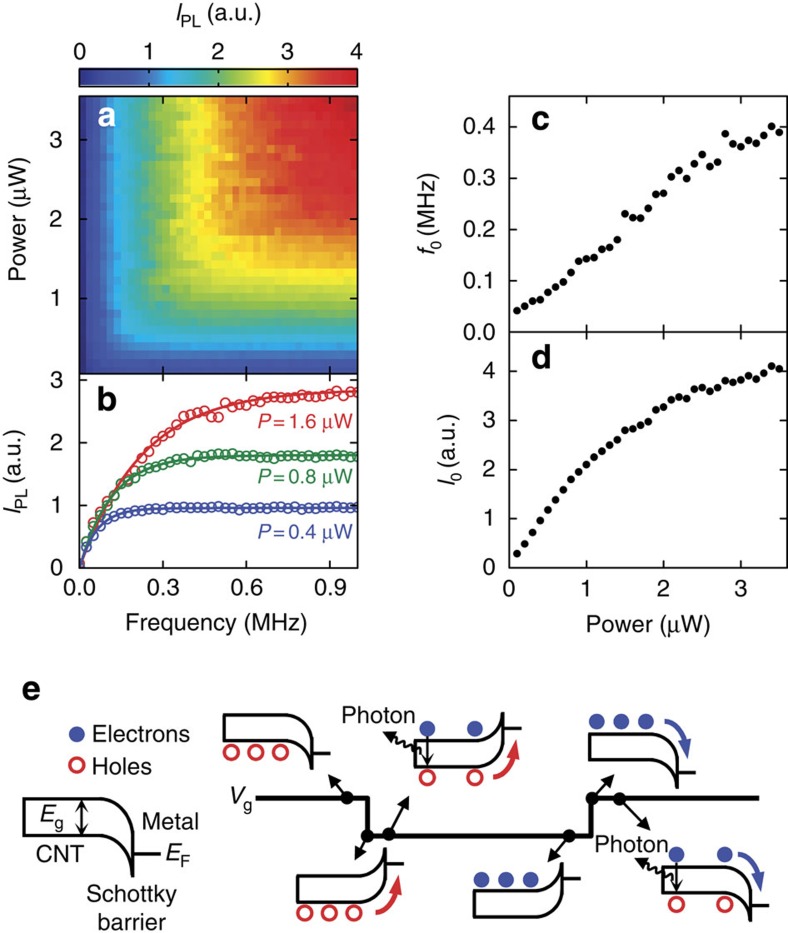
Frequency-domain measurements. (**a**) Integrated PL as a function of *f* and *P* for a device with a (10,8) nanotube. *V*_a_=−*V*_b_=3.0 V, and *I*_PL_ is obtained by integrating the PL spectra from *λ*_em_=1,400 to 1,450 nm. (**b**) Frequency dependence of integrated PL for *P*=0.4 μW (blue), *P*=0.8 μW (green) and *P*=1.6 μW (red). Circles are data and lines are fits. (**c**,**d**) Power dependence of *f*_0_ and *I*_0_, respectively. (**e**) Schematic showing a microscopic physical mechanism. Red open circles and blue filled circles represent holes and electrons, respectively. Short horizontal lines on the right side of the band diagrams indicate the Fermi level of the contact metal.

We further examine the PL recovery process in the frequency domain. In Fig. 3a, PL intensity is plotted as a function of the square-wave frequency and the excitation laser power for another device. Typical frequency dependence data are shown in Fig. 3b for three different excitation powers. *I*_PL_ recovers linearly at low frequencies and shows saturation at high frequencies, consistent with the observation in Fig. 2. Measurements up to higher frequencies have shown similar results (Supplementary Fig. 2). It is noteworthy that *I*_PL_ does not depend on the laser excitation power in the low-frequency regime, which implies that the emission intensity is limited by the number of voltage transitions. In addition, it can be seen that the saturation occurs at a higher frequency when the excitation power is increased.

To extract the characteristic saturation frequency *f*_0_, we fit the data to 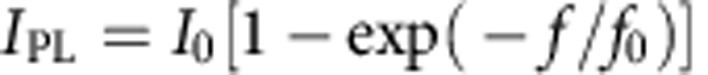, where *I*_0_ is the saturation intensity. As shown in Fig. 3b, we obtain good fits, and the results are summarized in Fig. 3c,d. The saturation frequency increases linearly with laser power, indicating that the photoexcited carriers are playing a role in the PL recovery process. The saturation intensity, which would be equivalent to the zero-voltage intensity, shows a sublinear behaviour known to be caused by efficient exciton–exciton annihilation in CNTs^21^,^24^,^25^,^26^,^27^.

In Fig. 3e, we propose a model that can explain the experimental observations, where photocarriers charge up the nanotube and voltage reversal causes discharging. Just before a transition from positive to negative gate voltage, photoexcited holes have accumulated in the nanotube. The photoexcited electrons are collected into the contact because of the band bending, while the Schottky barrier keeps the holes from escaping. In such a state, PL is efficiently quenched by the Auger process involving the carriers^12^,^13^,^14^,^15^,^16^. When the voltage is reversed, the Schottky barrier disappears and the holes are easily swept into the contact. Since the tube is now free of carriers, it becomes luminescent until the photoexcited electrons have accumulated enough to quench the PL in a self-limiting manner. Essentially, the voltage steps reset the nanotubes to become bright again. For the opposite polarity voltage transitions, the nanotube should also become temporarily luminescent because of the conduction and valence band symmetry^28^.

This model explains all of the key experimental features observed. PL recovery only occurs when the voltage transition passes through 0 V, as such a voltage sign reversal is required to sweep the carriers into the contact. Because nanotubes become luminescent for every voltage step, PL intensity is proportional to *f* in the low-frequency regime. At high frequencies, the reset happens before the carriers are accumulated, suppressing the quenching and recovering the PL emission to the zero-voltage level. Since photocarrier generation happens faster for higher powers, the linear power dependence of the saturation frequency can also be explained. Such a power dependence implies that carrier tunnelling from the Schottky contact^29^ is negligible compared with photoexcitation. We note that *f*_0_ approaches a non-zero value for the lowest powers in Fig. 3c and deviates from the linear relation, suggesting the contribution of carrier accumulation through the contacts.

### Gate-controlled pulse-train generation

**Figure 4:**
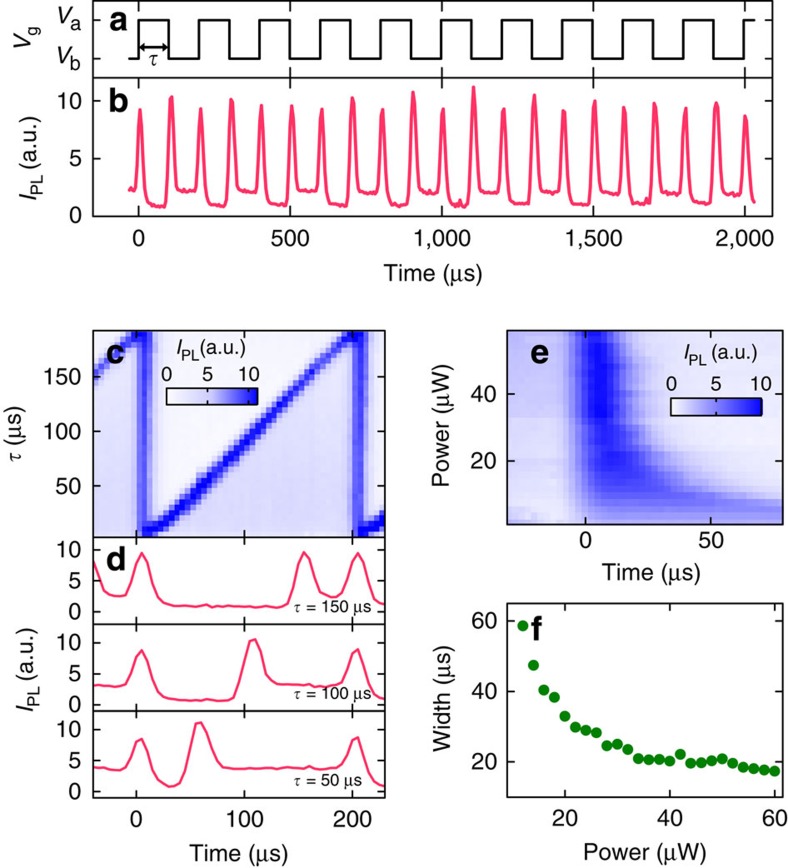
Time-domain measurements. A (13,3) nanotube is measured with *V*_a_=+1.2 V, *V*_b_=−1.8 V and *f*=5,011 Hz. The spectral integration window for *I*_PL_ is from *λ*_em_=1,440 to 1,490 nm. (**a**,**b**) Time dependence of gate voltage and integrated PL, respectively. (**c**) Integrated PL as a function of time and *τ*. (**d**) Temporal evolution of integrated PL for *τ*=50, 100 and 150 μs. For **b**–**d**, *P*=50 μW is used. (**e**) Integrated PL as a function of time and *P*. (**f**) Laser power dependence of pulse width.

If this model were correct, we expect pulsed light emission to occur just after the voltage transitions. We have performed time-domain measurements of the spectrally integrated PL on another device to directly observe such optical pulses. The results are shown in Fig. 4a,b, unambiguously confirming the existence of a series of pulses that are synchronized with the gate voltage transitions. The device effectively converts an electrical clock into an optical pulse train with twice the frequency, consistent with the microscopic physical mechanism proposed above.

As the optical pulses are generated by the voltage transitions, pulse timings can easily be controlled through the gate voltage waveform. In Fig. 4c,d, we present measurements performed for various time delay *τ* between the upward and the downward voltage steps. The pulse timing can be tuned continuously throughout the repetition period, suggesting that more complex sequences would be possible.

In addition to the timing, it is also possible to control the temporal width of the pulses through the excitation laser power. Because the pulse duration is determined by the carrier accumulation time, we expect pulse width to become narrower at higher powers. Such a control is shown in Fig. 4e, in which the power dependence of the PL intensity near the voltage step is plotted. At low powers, the pulse width is broad and the PL intensity is low. As the power is increased, the PL intensity becomes stronger, and at the same time the pulse width shortens significantly. We plot the full-width at half-maximum of the PL pulses in Fig. 4f, showing pulse narrowing to ~17 μs, which is limited by the time resolution of our measurement setup.

Such a change in the pulse width explains why the PL intensity does not depend on excitation power at low frequencies (Fig. 3a,b). If we assume that the emission rate is proportional to the excitation power, the time-integrated emission intensity per pulse should be independent of the laser power because the accumulation time is inversely proportional to the laser power, cancelling out the power dependence.

## Discussion

Our results demonstrate the potential for performing electrical-to-optical signal conversions at the nanoscale using individual nanotubes. A unique feature of the pulse-train generation presented here is that only a voltage step, rather than a voltage pulse, is needed to generate an optical pulse, implying that the optical pulse train can have a higher bandwidth than the electrical signal. Similar frequency conversion, though only through electrical signals, have been demonstrated using graphene devices^30^, underscoring the uniqueness of nanocarbon materials. In principle it should be possible to generate much shorter pulses if we place the nanotubes in vacuum, as it allows the excitation power to be increased by a few orders of magnitude.

## Methods

### Device fabrication

The devices are fabricated in a manner similar to suspended-CNT field-effect transistors^15^,^31^, but with silicon-on-insulator substrates with 260 nm of top silicon layer and 1 μm of buried oxide. The top silicon layer is boron doped with a resistivity of 18.0±4.5 Ω cm. We start by dry etching trenches through the top silicon layer, followed by a wet etch to further remove ~200 nm of buried oxide. The top silicon layer is thermally oxidized at 900 °C for 1 h to form a SiO_2_ layer with a nominal thickness of 20 nm. We then perform electron beam lithography to define the two metal pads, one each for contacting the nanotubes and the gate. The nanotube contact is placed right next to the trench, while the contact for the gate is located 5 or 10 μm away from the trench. For the electrodes, we evaporate 2 nm of Ti followed by 30 nm of Pt. From catalyst particles placed on the electrodes, nanotubes are grown over the trench onto the gate by chemical vapour deposition^32^,^33^.

### PL microscopy

The emission properties of devices are characterized using a confocal microspectroscopy system similar to that described in previous work^21^,^34^, but with an automated three-dimensional stage for scanning the sample instead of a laser-scanning mirror. The samples are excited with a wavelength-tunable continuous-wave Ti:sapphire laser, and PL is detected by an InGaAs photodiode array attached to a spectrometer. The excitation and detection spot sizes are ~1 and ~5 μm, respectively. The laser polarization angle is adjusted to maximize the PL signal, and the excitation wavelength is tuned to the *E*_22_ resonance unless otherwise noted. All measurements are done in air at room temperature.

### Time-resolved measurements

A function generator is used to generate a square-wave form with a rise/fall time of 20 ns, and an optical chopper phase-locked to the function generator is placed at the entrance of the spectrometer. The chopper has a duty cycle of ~6% giving a temporal resolution of ~13 μs at *f*=5,011 Hz, and the phase relative to the square-wave is scanned to obtain the time dependence.

## Author contributions

Y.K.K. conceived the experiments and supervised the project. M.J. fabricated the devices and performed the measurements. Y.K. and M.Y. assisted in optimizing the device fabrication processes. A.I. developed the microscopy system. T.S. captured the electron micrographs. M.J. and Y.K.K. analysed the data and prepared the manuscript. All authors discussed the results and commented on the manuscript.

## Additional information

**How to cite this article:** Jiang, M. *et al.* Gate-controlled generation of optical pulse trains using individual carbon nanotubes. *Nat. Commun.* 6:6335 doi: 10.1038/ncomms7335 (2015).

## Supplementary Material

Supplementary Information Supplementary Figures 1-2 and Supplementary Note 1.
