# Supplementary material for: Gate-controlled generation of optical pulse trains using individual carbon nanotubes
Source: Nat Commun. 2015 Feb 27;6:6335. doi: 10.1038/ncomms7335 (PMC4351562; doi:10.1038/ncomms7335)
Supplement: Supplementary Information — Supplementary Figures 1-2 and Supplementary Note 1. [file ncomms7335-s1.pdf]

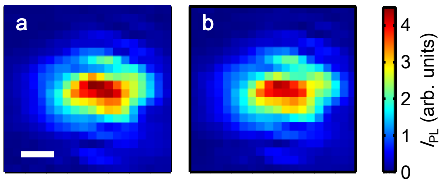

Supplementary Figure 1 | Imaging measurements. (**a**) Photoluminescence image taken at zero gate voltage. (**b**) Image of the same nanotube under square-wave gate voltage with V_a = 2.0 V and V_b = −2.0 V at f = 100 kHz. A nanotube with a chirality of (10,9) is measured under P = 10 μW and λ_{ex} = 872 nm. 50-nm wide spectral integration window centered at λ_{em} = 1515 nm is used for I_{PL}. Scale bar is 1 μm.

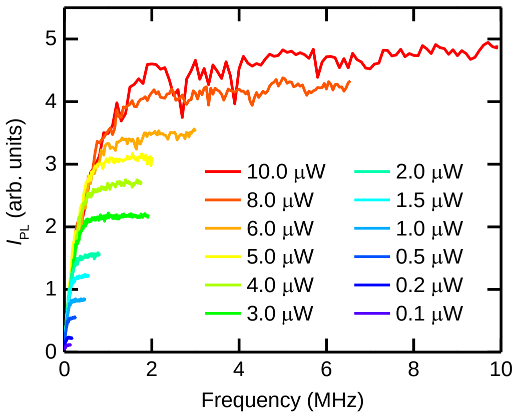

Supplementary Figure 2 | Higher frequency behavior. For another nanotube with a chirality of (9,8), frequency dependence measurements have been performed up to f = 10 MHz for V_a = 3.0 V and V_b = −3.0V. The spectral integration window for I_{PL} is from λ_{em} = 1360 nm to 1410 nm. λ_{ex} = 802 nm and P = 0.1 μW to 10.0 μW are used.

Supplementary Note 1

From the resistivity of 18.0 ± 4.5 Ω cm and the thickness of 260-nm for the top Si layer, sheet resistance becomes 690 kΩ. Capacitance to the substrate is 3.5 nF cm^{-2} for the oxide thickness of 1 μm with relative permittivity of 3.9 for SiO_2. For a gate dimension of 10 μm × 200 μm, the relevant resistance and capacitance are 35 kΩ and 69 fF, respectively, resulting in a capacitive cut-off frequency of 67 MHz.
